# Looking at the Mental Health of Children and Adolescents with Cleft Lip and/or Palate through Neuroticism and Emotional Regulation Strategies: A Case-Controlled Observational Study

**DOI:** 10.3390/jcm13113033

**Published:** 2024-05-22

**Authors:** Ana Ruiz-Guillén, María José González-Olmo, Esther Castañeda-López, Martín Romero-Maroto, Cecilia Peñacoba-Puente

**Affiliations:** 1 Department of Paediatric Dentistry, Rey Juan Carlos University, 28922 Alcorcón, Spain; ana.guillen@urjc.es; 2 Department of Orthodontics, Rey Juan Carlos University, 28922 Alcorcón, Spain; mariajose.gonzalez@urjc.es (M.J.G.-O.); martin.romero@urjc.es (M.R.-M.); 3 Department of Psychology, ESCUNI (Centro Universitario de Educación), 28047 Madrid, Spain; ecastaneda@escuni.es; 4 Department of Psychology, Rey Juan Carlos University, 28922 Alcorcón, Spain

**Keywords:** children and adolescents, cleft lip and palate, neuroticism, emotional regulation, mental health

## Abstract

**Background:** Children and adolescents with cleft lip and/or palate (CL/P) are at an increased risk of developing emotional disorders. This study aims to explore this question in greater depth by addressing three objectives: (1) the presence of neuroticism as an indicator of emotional symptomatology, (2) the use of adaptive and non-adaptive emotional regulation strategies, and (3) the relationship between these strategies and neuroticism. **Methods:** A case–control correlational methodology was employed, with 60 children and adolescents with CL/P (mean age = 12.80 years; 33 females) and 60 non-clinical equivalent children and adolescents. **Results:** The CL/P group has higher scores on neuroticism (*t* = −7.74; *p* ≤ 0.001, d Cohen = 1.43) and lower scores in almost all emotional regulation strategies. The presence of CL/P moderated the relationship between neuroticism and self-blame (Beta = −0.46, *t* = −2.81, *p* = 0.005), rumination (Beta = −0.49, *t* = −3.73, *p* < 0.001), catastrophizing (Beta = −0.61, *t* = −4.26, *p* < 0.001), and blaming others (Beta = −0.45, *t* = −2.84, *p* = 0.005). This model predicted a significant variance of neuroticism (all *p* < 0.005), which ranged from 39% to 41%. **Conclusions:** The CL/P group has worse mental health indicators. Particularly novel results about the CL/P group are the lower scores on regulation strategies (both adaptive and non-adaptive) and the fact that non-adaptive strategies contribute, contrary to their effect in the general population, to a decrease in neuroticism. It supports the need to incorporate mental health indicators in the diagnosis and treatment of children and adolescents with CL/P.

## 1. Introduction

Cleft lip/palate (CL/P) is a type of congenital disorder affecting approximately 1 in 700 newborns caused by embryonic defects in the formation of the upper lip and palate during the early stages of pregnancy [1]. The etiology is unknown, although associations with racial variations, environmental exposures, and socioeconomic status have been found [2]. The comorbidity associated with this disorder often requires multidisciplinary treatment involving speech therapy, psychological therapy, dental restorations, orthodontic, and surgical treatment throughout the child’s development [1,2]. Functional defects and psychological consequences in these patients can extend into adolescence and last into adulthood [3].

The degree of visibility of this medical condition may be associated with mental health problems [4], as appearance plays a critical role in social interactions [3,5]. The presence, type, and severity of psychosocial difficulties may change across development, suggesting that children with CL/P may be more likely to experience psychosocial difficulties from middle childhood through adolescence [6]. The relevance of understanding and meeting the mental health needs of young people is reinforced by the growing recognition of the importance of the early years in future mental health problems in adulthood [7]. As observed among the general population, experiencing one or more traumatic events in childhood may increase vulnerability to developing psychopathology as a young adult after stress exposure [8]. Children show individual differences in behavioral style and temperament from birth onward, and these individual differences play a significant role in the course of normal and deviant psychological development, at least into early adulthood [9]. This research points to neuroticism and non-adaptive emotional regulation strategies as indicators of poor mental health [8,9]. Therefore, in order to evaluate the mental health of children and adolescents with craniofacial alterations that condition their interaction with the environment and, consequently, their behavior, it will be important to analyze those psychological variables (i.e., emotional regulation and neuroticism) that may interfere in their correct development and which may influence their mental health.

Emotional regulation is a multi-faceted construct involving the experience and differentiation of emotions, both pleasant and unpleasant, as well as the ability to modulate them, which is acquired during development. It is a process that people use to monitor, evaluate, and modify emotions to meet goals [10,11], including aspects that arise both within an individual and between the individual and his or her environment [10]. Responses to emotional events during childhood will contribute to the development of skills or strategies that allow emotion to be regulated in an adaptive or non-adaptive manner [12].

Individuals who frequently use non-adaptive strategies (i.e., rumination, self-blame, blaming others, and catastrophizing) are at risk of developing psychological disorders such as depression and/or anxiety [10], whereas the use of adaptive strategies (i.e., positive refocusing, positive reappraisal, putting into perspective, planning, and acceptance) contribute to better tolerance and management of emotions associated with negative experiences [12]. Thus, difficulties in emotional regulation and the use of non-adaptive strategies are related to the development of emotional disorders (i.e., anxiety and depression), emotional instability, and negative affectivity (i.e., neuroticism), which in turn, through feedback processes and vicious cycles, contribute to the maintenance and chronification of these disorders [13].

Within such mental health implications, and according to the Big Five model [14], neuroticism is characterized by an individual’s tendency to experience negative affect in response to stress, which is related to general distress [15]. Positive associations of neuroticism have been found between symptoms of depression and anxiety [15,16,17]. Likewise, different research studies highlight the association between the use of emotional regulation strategies and neuroticism. Thus, neuroticism is associated with greater use of non-adaptive emotional regulation strategies [13,18] and less use of cognitive reappraisal [19], a strategy conceptualized as adaptive.

Therefore, since non-adaptive emotional regulation leads to emotional disorders, including anxiety, depression [10,13,18], and neuroticism [15,20], it is important to investigate the influence of the management of these cognitive processes of emotional regulation in patients susceptible to emotional disorders, such as children and adolescents with CL/P, assessing in this case as an indicator of emotional disorder, their emotional instability (i.e., neuroticism). This type of research is particularly relevant since neuroticism, in turn, biases attentional focus towards states of emotional stress that interfere with cognitive performance, leading to non-adaptive behaviors and a lack of coping skills [21]. Thus, people with high neuroticism use more non-adaptive strategies, are less involved in reappraisal to regulate their emotions, and have a lower ability to reduce negative emotions [19]. Individual differences in emotional reactivity and in the ability to effectively modulate emotional responses have significant implications for healthy psychological function and mental well-being [22]. Such processes ultimately contribute to the chronification of emotional disorders through the establishment of vicious circles and feedback processes.

Despite there being enough conclusive evidence about psychological problems in children and adolescents with CL/P [5,6], the scarcity of studies analyzing the role of neuroticism and emotional regulation strategies in this population is particularly relevant. The aim of this study was to assess the relationships between these variables to gain a more comprehensive understanding and with the strength of including a control group. Specifically, three specific aims are proposed in children and adolescents with CL/P: (1) to analyze the presence of neuroticism as an indicator of emotional symptomatology, (2) to explore the use of adaptive and non-adaptive emotional regulation strategies, and (3) to analyze the relationship between emotional regulation strategies and neuroticism. In particular, a higher neuroticism score is hypothesized in children and adolescents with CL/P compared to the group of children and adolescents without CL/P. Regarding emotional regulation mechanisms, lower scores on adaptive strategies and higher scores on non-adaptive strategies are expected to be found in the CL/P group. Finally, having a cleft was anticipated to mediate the relationship between emotional regulation and neuroticism, as it is expected that there is some dysfunction in these variables during the stages of psychological development and the beginning of their social interactions.

## 2. Materials and Methods

### 2.1. Settings and Participants

A cross-sectional study was carried out with a sample composed of 60 children and adolescents with CL/P (M_age_ = 12.80 years; SD = 2.79; 27 males and 33 females; age range = 8–18 years) who were recruited from two orthodontic dental clinics specializing in cleft treatment, as well as an equivalent sample in terms of sex, race, age, ethnicity, and socioeconomic status of 60 non-clinical children and adolescents from the same geographical areas in their usual dental clinic (M_age_ = 12.16 years; SD = 2.06; 32 males and 28 females). Study data were collected from 2016 to 2019. 

The inclusion criteria for both groups were to be between 10 and 18 years old and not to have any developmental and/or intellectual impairment. In addition, children with additional conditions to CL/P in the clinical group were not included in this study (i.e., patients with a syndrome-associated cleft were excluded). Data were collected via self-report questionnaires for each child, with a format adapted to the age of the participants. Given the wide age range of the sample, it was divided into two groups to avoid possible comprehension biases. The first group was those under 12 years of age, who received help from the researcher to complete the questionnaires, explaining the items that were complicated to understand through examples without interfering with their answers. The second group was patients aged 12 years and older, in which the questionnaire was self-administered. The first group consisted of 25 (41.6%) children with a cleft and 26 (43.3%) children without a cleft. The 12- to 18-year-old group consisted of 35 (58.4%) children with a cleft and 34 (56.7%) children without a cleft. Parents of the participants and the participants themselves received oral and written information, and a researcher was available to answer questions about the survey. Informed consent was obtained from the patient’s parents or the patients themselves if they were 18 years old. After signing informed consent and answering their questions about the research, they were given the questionnaires. 

This study was conducted in accordance with the Declaration of Helsinki and followed the guidelines of the Research Ethics Committee of Rey Juan Carlos University (110720166716; 26 September 2016). 

### 2.2. Measures

Neuroticism: Neuroticism was measured with The Big Five Questionnaire for Children (BFQ-NA; [14]). This scale was validated for the Spanish population from 8 to 16 years old by Del Barrio et al. [23]. Neuroticism involves the individual’s difficulty in coping with stressful situations and is defined by items referring to irritability, anger, sadness, anxiety, worry, hostility, and lack of self-consciousness. To assess neuroticism, the BFQ-NA contains 13 items rated on a 5-point Likert scale. Higher scores indicate greater neuroticism.

Cognitive Emotional Regulation: The child version of the Cognitive Emotion Regulation Questionnaire (CERQ-k; [24]) was used. Specifically, the Spanish version of CERQ-k was administered [25]. The CERQ-k is composed of 36 items on a 5-point Likert-type response scale ranging from 1 (never) to 5 (always). It allows the assessment of nine emotion regulation strategies (with four items each) through questions about negative or uncomfortable events. The strategies are grouped into adaptive and non-adaptive strategies. Adaptive strategies include positive refocusing (i.e., thinking about more pleasant topics instead of thinking about the current negative situation), planning (i.e., thinking about what steps to take and how to handle negative events), positive reappraisal (i.e., thoughts that give positive meaning to events in terms of personal growth), and putting into perspective (i.e., downplaying the seriousness of events or emphasizing their relativity in comparison to other events). Non-adaptive strategies include self-blame (i.e., thoughts of blaming oneself for what has been experienced), rumination (i.e., thinking about feelings associated with negative events), catastrophizing (i.e., emphasizing the theme of an experience generating emotional distress), and blaming others (i.e., placing the blame for what has been experienced on others). Finally, acceptance, as assessed in the instrument, is not clearly shown to be an adaptive or non-adaptive strategy as it includes elements of both acceptance and resignation. Total scores are obtained by summing separately all strategies, with higher scores representing a more frequent use of the strategies.

Finally, all participants were asked questions about sex (male, female) as well as the educational level of both parents according to the International Standard Classification of Education (ISCED) [26]. In addition, members of the CL/P group were asked about the diagnosis of CL/P (cleft lip, cleft palate, or cleft lip and palate), whether they had undergone a second surgery (yes/no), age at first surgery (months) and age at second surgery (months).

### 2.3. Statistical Analysis

SPSS Statistics V21.0 was used to perform all analyses. Both descriptive and internal consistency analyses (Cronbach’s alpha) were performed. First, descriptive and bivariate Pearson correlation analyses between neuroticism and emotional regulation subscales were performed. To analyze possible differences in the variables assessed between CL/P and non-CL/P groups, *t*-tests were carried out. To calculate the effect size, Cohen’s d was used. According to Cohen [27], small Cohen’s d values are about 0.2, medium ones about 0.5, and high ones about 0.8. Finally, moderation analyses were performed using model 1 of PROCESS Macro version 3.4 [28]. The presence of CL/P was used as the moderator (yes/no), emotional regulation strategies as the independent variables, and neuroticism as the outcome. Eight models were tested (considering all strategies except for acceptance), one for each dimension of emotional regulation (self-blame, rumination, positive refocusing, planning, positive reappraisal, putting into perspective, catastrophizing, and blaming others). To increase the robustness of the standard errors of parameter estimates, bootstrapping was applied to examine the significance of all effects using 95% bias-corrected confidence intervals of these effects based on 5000 bootstrapped samples. When the confidence interval does not contain zero, the results are considered statistically significant.

## 3. Results

### 3.1. Characteristics of the Sample

The CL/P group presented a mean age of 12.81 ± 2.79, similar to that observed in the group without CL/P (mean = 12.16 ± 1.19), with no significant differences between the two groups (*t* = 1.61, *p* = 0.109). The CL/P group consisted of 27 boys and 33 girls, with no statistically significant differences compared to the distribution of the non-CL/P group (χ2 = 0.875, *p* = 0.349). No significant differences are found between the two groups (with and without CL/P) in the educational level of fathers (χ2 = 1.237, *p* = 0.592) and mothers (χ2 = 1.137, *p* = 0.472). Following the International Standard Classification of Education (ISCED) (UNESCO, 2017), the highest percentages of fathers of children and adolescents with CL/P are placed at level 4 post-secondary non-tertiary education (44.8%), followed by level 7: master’s or equivalent (36.2%). Approximately 20% of the fathers are at level 1: primary education or the first stage of basic education. Regarding the educational level of the mothers of children and adolescents with CL/P, 25% of the total is at level 1, 37% at level 4, and 37% at level 7. Families of both groups were predominantly of middle socioeconomic status, with no statistically significant differences between them (*p* = 0.532). 

Regarding the clinical characteristics of the CL/P group, 10 of the children/adolescents had cleft lip, 14 cleft palate, and 36 cleft lip and palate. Almost all of them (90%) had already received lip and palate treatment, and 90% of them had already undergone a second surgery. Patients with a cleft reported that the psychological therapy they had engaged in had been occasional. This is a treatment that is not included in the Spanish public health system for these patients, and if psychological support is needed, patients need to finance the expenses themselves.

### 3.2. Differences between the Two Groups (with and without CL/P) in Neuroticism and Emotional Regulation Strategies

Table 1 shows the differences in neuroticism and emotional regulation strategies between children and adolescents with and without CL/P. Children and adolescents with CL/P show higher scores in neuroticism (*t* = −7.74; *p* ≤ 0.001, d Cohen = 1.43). In relation to cognitive emotional regulation, significant differences can be observed in all strategies except for positive reappraisal and blaming others. In all strategies (both adaptive and non-adaptive), lower scores are observed in the group with CL/P (compared to the non-CL/P group): self-blame (*t* = 3.04; *p* = 0.003; d Cohen = 0.560); rumination (*t* = 3.31; *p* = 0.001; d Cohen = 0.615); positive refocusing (*t* = 2.89; *p* = 0.005; d Cohen = 0.538); planning (*t* = 2.69; *p* = 0.008; d Cohen = 0.501); putting into perspective (*t* = 2.07; *p* = 0.040; d Cohen = 0.386); and catastrophizing (*t* = 3.14; *p* = 0.002; d Cohen = 0.583).

### 3.3. Multivariate Linear Regression and Moderation Analyses

Before performing the regression analyses and in order to control for covariates, bivariate analyses were carried out between the variables under study (i.e., emotional regulation strategies and neuroticism) and sex and age in each of the groups (with and without a cleft). The following was observed:

(1) In the group with a cleft, statistically significant relationships were only identified between age and self-blame (R^2^ = 0.347, *p* = 0.009).
(2) In the group without a cleft, no significant relationship was identified between the variables under study, neither with age nor with sex.

Based on these results, age is introduced in regression analyses when trying to predict neuroticism from self-blame, including CL/P as a moderator. 

Table 2 shows the results of the regression analyses (from emotional regulation strategies to neuroticism), including CL/P as a moderator. In almost all cases, through the different emotional regulation strategies, a significant direct effect of having CL/P in neuroticism is observed, except for positive refocusing and planning. 

Direct effects of the following emotional regulation strategies are also observed: rumination, positive refocusing, catastrophizing, and blaming others. Rumination, catastrophizing, and blaming others have a positive effect on neuroticism, whereas positive refocusing has a negative effect on it.

Regarding the moderation analyses, the results revealed that CL/P moderated the relationship between self-blame and neuroticism (Beta = −0.42, *t* = −2.44, *p* = 0.016), rumination and neuroticism (Beta = −0.49, *t* = −3.73, *p* < 0.001), catastrophizing and neuroticism (Beta = −0.61, *t* = −4.26, *p* < 0.001), and blaming others and neuroticism (Beta = −0.45, *t* = −2.84, *p* = 0.005). This model predicted a significant variance of neuroticism (all *p* < 0.005), which ranged from 39% to 41%.

**Figure 1:**
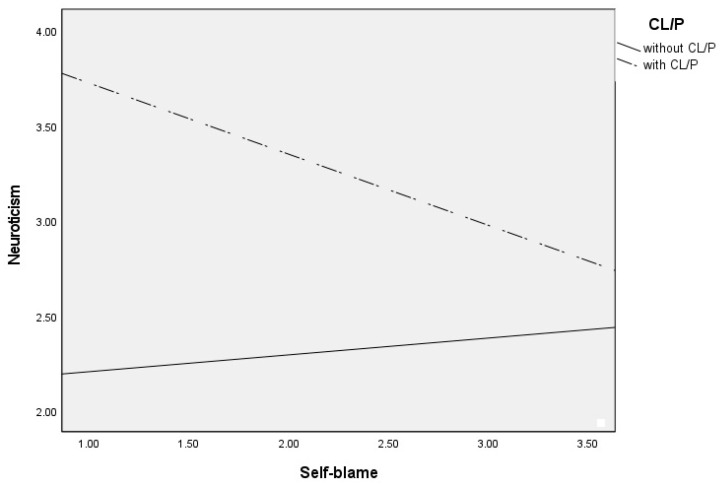
Differential effects between self-blame and neuroticism depending on CL/P and without CLP group.

**Figure 2:**
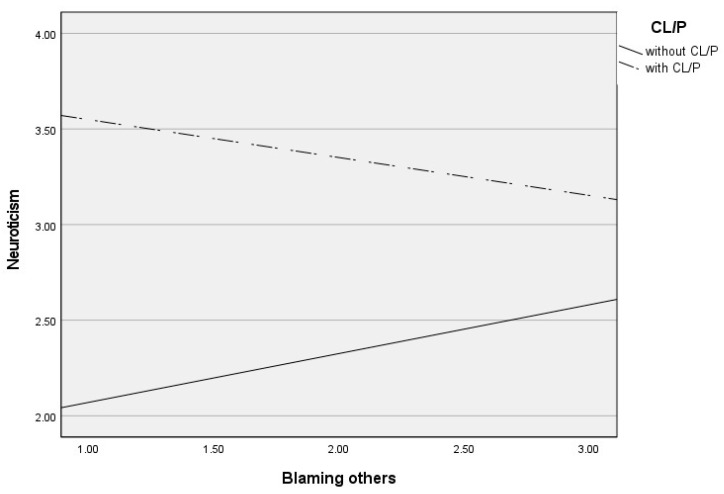
Differential effects between blaming others and neuroticism depending on CL/P and without CLP group.

**Figure 3:**
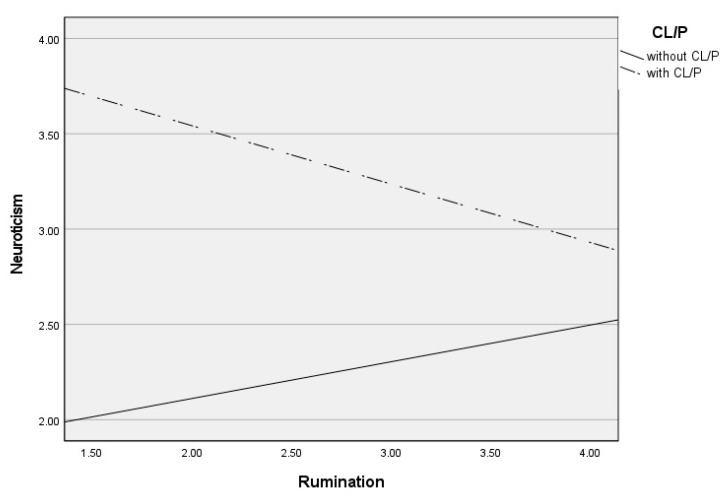
Differential effects between rumination and neuroticism depending on CL/P and without CLP group.

**Figure 4:**
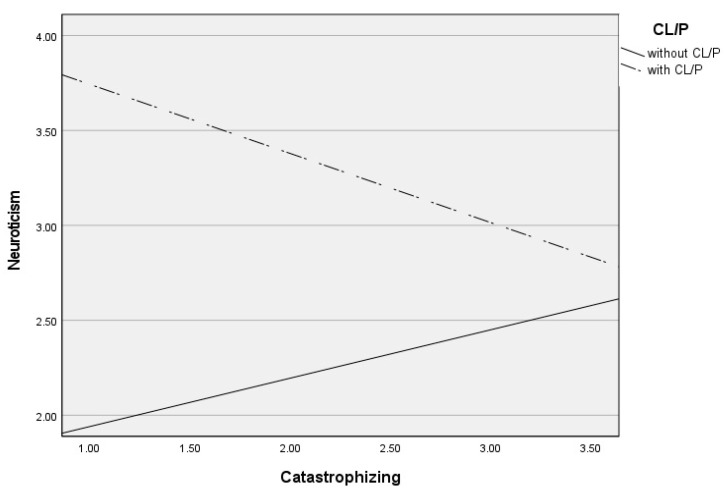
Differential effects between catastrophizing and neuroticism depending on CL/P and without CLP group.

Post hoc analyses were used to analyze significant moderations in greater depth and are presented in Table 3. These analyses revealed that self-blame contributed to increased neuroticism only in CL/P patients (see Figure 1), blaming others contributed to increased neuroticism only in children and adolescents without CL/P (see Figure 2), and rumination (see Figure 3) and catastrophizing (see Figure 4) contributed to increased neuroticism in both groups. 

## 4. Discussion

This research aimed to investigate the mental health of children and adolescents with cleft lip/palate through neuroticism, emotional regulation strategies, and the relationship between them, comparing the results with a group of children and adolescents without CL/P. Our first objective is to assess whether the levels of neuroticism in children and adolescents with CL/P differ from the levels in children and adolescents without CL/P. Despite the lack of research in this regard, higher neuroticism scores were expected in the CL/P group, taking into account previous studies that report greater emotional symptomatology, specifically anxiety and depression, in patients with CL/P, specifically in children and adolescents [5,6,29], and the well-documented association between neuroticism and anxiety and depression [16,30]. Although there are, to our knowledge, no studies examining neuroticism in this population, numerous studies in other clinical populations report higher levels of neuroticism than in the general population. This is the case of studies carried out in dyslexia [31], burns [32], heart disease [33], or epicrania fugax [34]. Specifically in children and adolescents, higher neuroticism scores have also been observed in blind [35] and asthmatic children [36].

The second objective of our research focused on the analysis of the emotional regulation strategies of children and adolescents with CL/P. Our results show statistically significant differences between both groups (with and without CL/P) in all strategies, except for positive reappraisal and blaming others, with the CL/P group showing lower scores in all cases. These results, given the lack of previous literature on the matter, show interesting findings that would require further research. Our hypothesis in this regard expected to find higher scores on non-adaptive strategies and lower scores on adaptive strategies in children and adolescents with CL/P. Despite the absence of previous research, this hypothesis was put forward on the basis of the relationship established in the literature in the general population of children and adolescents between non-adaptive regulation strategies and emotional disorders on the one hand and between adaptive regulation strategies and psychological well-being on the other [12,21]. In this line of research, Rodríguez-Menchón et al. [37], when analyzing emotional regulation in a group of children and adolescents from the general population, found that children with anxious symptoms tended to present a greater use of maladaptive regulation strategies, especially self-blame, rumination, and catastrophizing, compared to children without anxious symptoms. However, the results in children and adolescents with CL/P partially confirm our hypothesis. The group with CL/P (in contrast to the group without CL/P) presents lower scores in adaptive but also in non-adaptive strategies. 

Regarding the lower scores found in the non-adaptive strategies, the results are consistent with those found by Lecommandeur et al. [38], using the same instrument in youths aged 7.5 to 16 born with unilateral CL/P. To our knowledge, this is the only study on emotional regulation strategies carried out in this population. Given the absence of previous research, these low scores in non-adaptive regulation strategies, which in principle would be adaptive and healthy, could be related to resilience, a dynamic process by which individuals mobilize available resources to recover from adversity. A resilient individual might have a broader mindset and a more flexible cognitive process, employing less non-adaptive strategies to promote emotional stability and weaken negative emotions caused by neuroticism [39]. Some studies have shown that children with CL/P are particularly resilient in their management of negative affect, which may be due to early exposure to physical and emotional challenges, which allows them to cope more successfully than children without CL/P to ordinary stressors [40]. It has also been reported in other studies [41] that adolescents with a visible cleft present better functioning than adolescents without CL/P, in addition to presenting fewer symptoms of depression, indicating an emotional resilience, which could be caused by better development of skills to cope with the consequences of stress. However, this explanatory hypothesis of resilience would not justify the lower scores also found in the adaptive regulation strategies in our study.

Thus, taken as a whole (low scores in both adaptive and non-adaptive strategies), the poor development of regulation strategies in children and adolescents with CL/P could be considered. Specifically, van Dalen et al. [4] alluded to the excessive protection of these children by their parents, which could justify the low use of emotional regulation strategies by the child found in our research. Another possible explanation for these lower scores in emotional regulation strategies could be found in the potential victimization of children and adolescents with a cleft, given the lower development of self-blame found in our study. Previous research [42] shows that victimized children use a variety of ineffective and incoherent strategies, proving emotional dysregulation [43]. These hypotheses about the difficulty of regulating emotions due to the lack of both adaptive and non-adaptive strategies would be compatible with the higher scores found in alexithymia in this population compared to the non-clinical population [44]. 

Our third objective (modulation) aimed at assessing the relationship between regulation strategies and neuroticism, taking into account the role of having CL/P. Analyzing the interaction results, the differential effect of emotional regulation strategies in children and adolescents with and without CL/P is revealed. Thus, the results found in the group without CL/P show the expected relationship between emotional regulation strategies and neuroticism. Specifically, the use of non-adaptive strategies such as blaming others, rumination, and catastrophizing contribute to increasing levels of neuroticism, consistent with the existing literature [11,13,20]. Children with neuroticism show more depressive symptoms, observing the role of non-adaptive strategies, such as rumination and catastrophizing, between neuroticism and depression [12]. Likewise, higher levels of rumination, catastrophizing, and blaming others are associated with greater symptoms of depression, anxiety, and emotional instability [18,30]. 

Particularly novel results are observed in the relationship between emotional regulation strategies and neuroticism in children and adolescents with CL/P. Contrary to what happens in the general broader population [11,13,20], our results indicate that non-adaptive strategies contribute to reducing neuroticism in children with CL/P. Specifically, higher levels of self-blame, rumination, and catastrophizing are associated with lower levels of neuroticism. To our knowledge, there are no studies in this population that analyze this relationship. In line with what was previously mentioned [4,42], it could be hypothesized that in children and adolescents with CL/P, intrusive and negative thoughts could contribute to the resignation of their identity as a way of understanding the unpleasant emotion they are experiencing as an inherent part of their condition of CL/P. This would contribute, in turn, to reducing levels of neuroticism. Further research is necessary in this regard. The innovative outcomes may carry important implications for the focus and content of intervention and prevention of mental health of children and adolescents with CL/P. Because the ability to regulate emotions and think flexibly can help cope with neuroticism [11], psychological interventions in these patients should be directed at emotional regulation. From a clinical point of view, investigating the relationships between the variables that influence psychological well-being will help these patients overcome the risks derived from their condition. Similarly, it would be of great interest for future lines of research to carry out an analysis of these variables after receiving psychological therapy in order to check the evolution of these patients.

### Limitations

This study has some limitations. Firstly, due to its cross-sectional nature, no cause–effect relations could be established. Furthermore, the correlational design prevented us from establishing the origin and consequence (emotional regulation and neuroticism). Secondly, this investigation solely relied on children’s and adolescent’s self-reports; the detection of emotional regulation and neuroticism had to be made based on self-reported evaluation, which may have caused some biases. Thirdly, a convenience sample of children and adolescents with CL/P, obtained from two specialized centers, was used so it could limit the generalizability of the results. Finally, large cleft population studies are needed, in which individuals are analyzed according to the type of cleft present (cleft lip, cleft palate, or CL/P).

## 5. Conclusions

Children and adolescents with CL/P present greater emotional instability and less use of both adaptive and non-adaptive emotional regulation strategies. Furthermore, the results show that in this population (in contrast to what is observed in children without CL/P), rumination, catastrophizing, and self-blame contribute to the decrease in neuroticism.

## Figures and Tables

**Table 1:** Differences in neuroticism and emotional regulation strategies (with and without CL/P) (n = 120).

	α	Totals Mean (SD)	CL/P Mean (SD)	Controls Mean (SD)	*t*	*p*	d Cohen
Neuroticism	0.80	2.83 (0.90)	3.38 (0.73)	2.32 (0.73)	−7.74	˂0.001	1.43
Self-Blame	0.77	2.15 (0.88)	1.90 (0.72)	2.38 (0.96)	3.04	0.003	0.56
Rumination	0.80	2.82 (1.02)	2.51 (0.97)	3.11 (0.98)	3.31	0.001	0.615
Positive refocusing	0.76	3.15 (1.08)	2.86 (1.12)	3.42 (0.97)	2.89	0.005	0.538
Planning	0.77	3.42 (1.02)	3.16 (1.09)	3.66 (0.89)	2.69	0.008	0.501
Positive reappraisal	0.76	2.94 (1.07)	2.90 (1.06)	2.97 (1.09)	0.342	0.733	--
Putting into perspective	0.76	3.15 (1.08)	2.94 (1.13)	3.35 (0.99)	2.07	0.041	0.386
Catastrophizing	0.70	2.25 (0.94)	1.98 (0.81)	2.51 (0.99)	3.14	0.002	0.583
Blaming others	0.78	1.91 (0.84)	1.82 (0.82)	2.00 (0.86)	1.13	0.258	--

α: Cronbach’s alpha for the scale; SD (standard deviation).

**Table 2:** Prospective prediction of neuroticism from emotional regulation strategies and their interaction in CL/P group.

	R^2^	F	*p*	Beta	*t*	*p*	95% CI
DV = Neuroticism	0.39	18.27	<0.001				
Self-Blame				0.08	0.89	0.37	−0.10, 0.27
CL/P (yes/no)				1.91	5.13	<0.001	1.17, 2.65
Interaction				−0.42	−2.44	0.016	−0.75, −0.08
Age				−0.03	−0.95	0.341	−0.09, 0.03
DV = Neuroticism	0.41	26.96	<0.001				
Rumination				0.19	2.09	0.03	0.01, 0.37
CL/P (yes/no)				2.43	6.10	<0.001	1.64, 3.22
Interaction				−0.49	−3.74	<0.001	−0.76, −0.23
DV = Neuroticism	0.37	22.83	<0.001				
Positive Refocusing				−0.24	−2.50	0.013	−0.43, −0.05
CL/P (yes/no)				0.19	0.43	0.671	−0.68, 1.05
Interaction				0.25	1.98	0.052	−0.0001, 0.51
DV = Neuroticism	0.36	21.22	<0.001				
Planning				−0.18	−1.76	0.07	−0.40, 0.02
CL/P (yes/no)				0.38	0.76	0.45	−0.61, 1.38
Interaction				0.18	1.31	0.19	−0.09, 0.46
DV = Neuroticism	0.36	21.34	<0.001				
Positive Reappraisal				−0.14	−1.69	0.09	−0.32, 0.02
CL/P (yes/no)				0.81	2.03	0.04	0.02, 1.59
Interaction				0.08	0.65	0.51	−0.16, 0.33
DV = Neuroticism	0.35	20.49	<0.001				
Putting into perspective				−0.06	−0.71	0.47	−0.25, 0.12
CL/P (yes/no)				1.11	2.55	0.01	0.25, 1.97
Interaction				−0.02	−0.20	0.84	−0.28, 0.23
DV = Neuroticism	0.43	28.92	<0.001				
Catastrophizing				0.25	2.83	0.005	0.07, 0.43
CL/P (yes/no)				2.42	7.04	<0.001	1.74, 3.11
Interaction				−0.61	−4.26	<0.001	−0.90, −0.33
DV = Neuroticism	0.39	23.92	<0.001				
Blaming Others				0.25	2.36	0.01	0.04, 0.46
CL/P (yes/no)				1.93	5.81	<0.001	1.27, 2.59
Interaction				−0.45	−2.84	0.005	−0.76, −0.13

**Table 3:** Conditional effects of emotional regulation strategies on neuroticism depend on having CL/P or not.

CL/P	Beta (Self-Blame)	*t*	*p*	95% CI
No	0.086	0.89	0.37	−0.10, 0.27
Yes	−0.33	−2.35	0.020	−0.61, −0.05
CL/P	Beta (rumination)	*t*	*p*	95% CI
No	0.19	2.09	0.038	0.01, 0.37
Yes	−0.30	−3.16	0.002	−0.49, −0.11
CL/P	Beta (catastrophizing)	*t*	*p*	95% CI
No	0.25	2.83	0.005	0.07, 0.43
Yes	−0.36	−3.19	0.001	−0.58, −0.13
CL/P	Beta (blaming others)	*t*	*p*	95% CI
No	0.25	2.36	0.019	0.41, 0.46
Yes	−0.19	−1.68	0.094	−0.43, 0.03

CI: confidence interval.

## Data Availability

Data will be made available upon request.
